# Idiosyncrasy

**Published:** 1884-11

**Authors:** J. T. Kent

**Affiliations:** St. Louis


### IDIOSYNCRASY.

Prof. J. T. Kent, A. M., M. D., St. Louis.

This term has been used in allopathic nomenclature to define a condition supposed to be a special hypersensitive state always present in a particular patient. It is well known that some patients have an increased irritability for certain drugs. This susceptibility has been called, for want of a better explanation, an idiosyncrasy. It is a peculiarity of many people, so supposed; but, as a matter of fact, every person has some idiosyncrasy or peculiar susceptibility to something. Cases are on record of most striking susceptibility to certain poisons and noxious gases. In California, a child four years old was poisoned with four drops of Laudanum. I have seen dangerous symptoms follow a single drop of Aconite in an adult lady. I know a large, robust woman who becomes stiff and rigid in her joints whenever she inhales sewer gas or air from a common country closet vault. A man lately reported a case to me for advice at Monson & Co.'s pharmacy—a traveling man—who stated that whenever he slept in a room where Persian insect powder had been used he broke out with patulous eruptions and became a great sufferer. I once knew a man who would suffer greatly from the mere trace of Camphor that he would accidentally inhale in spite of himself in his ordinary travels. I am acquainted with a physician who dare not take a teaspoonful of custard, or he will have a diarrhœa in less than two hours.

A practicing physician told me years ago that he could not carry Rhubarb in his saddle-bags, as it always gave him a diarrhœa soon after inhaling it. We have people among us who are made sick by the commonest articles of the dinner-table. I presume every person has observed this peculiar idiosyncrasy. Why did not some wise man in allopathic medicine explain this, and not leave it for Hahnemann to solve by the law of similars? How simple that the similar power or force should create within the body such a turmoil. Were there no idiosyncrasy there would be no disease. This susceptibility being present, the noxious agent, though a million times too small for the microscope to reveal, will do its work and bring on disease, and even death, and the wonderfully wise pathologist has not solved the etiology or the method of this active destruction upon its medicine.

In drug proving we find a single dose of a drug exerting its power upon one prover, and the others escape until after having taken many doses or taken it many days. The highest potencies affect some provers, and large doses of the tincture are required to influence others.

Homœopathicity is almost, if not quite, identical with idiosyncrasy. A patient of mine said I must not give him anything with Strychnia in it, because the smallest doses of that drug made him worse, but his remedy was Nux vomica, which he detected in the second potency and declared it was Strychnia. It cured him permanently. It is fortunately a fact that our crude prescribers seldom make a close selection, or they would do a world of harm. The sensitiveness of a sick nerve to a homœopathic agency is wonderful, while the subject may bear a great amount of inappropriate crude drugging without apparent distress. It is more than likely that the four-year-old child that was poisoned with four drops of Laudanum would have found its remedy in potentized Opium on a single pellet No. 20. This idiosyncrasy can be produced by medicinal substances; thus, the provers of Thuja may get a diarrhœa after eating onions; the provers of Colch. are made sick by the smell of eggs; Plumbum provers cannot eat fish; Lycop. provers cannot eat oysters; while Ignatia provers are made sick by eating sugar and sweets; not that all suffer in this way, but many. These peculiar idiosyncrasies are also cured by the corresponding remedy. Many times have I cured with Thuja the peculiar diarrhœa brought on every time the patient eats onions. What explanation has our learned pathologist for this state? Can he by his wisdom cure it? No; his good patients go on suffering from their peculiar constitutional wrongs, and the good old doctor consoles him or her by soothing words or a dose of Opium or chalk mixture.

All there is of medicine that can permanently benefit man has come through the philosophy of Hahnemann; and this great stumbling-block of regular physicians (?), the idiosyncrasy, has become the keystone of scientific medicine, and explains itself when the philosophy of Hahnemann is understood.

The marked idiosyncrasy is not always observed for the crude materials, as is well known to all Hahnemannians, an instance of which is observed where crude common salt will not produce the slightest disturbance, although the patient craves and takes it largely in food, but the higher potencies produce the sharpest aggravation. The same may be observed with lime salts when there is a marked bone-salt inanition. Therefore, in cases where Lime is the remedy and lime-water is administered, not the slightest medicinal effect is produced; but the higher potencies act curatively, after which the corresponding saline is appropriated from the natural source, the food eaten.

This extreme susceptibility, called idiosyncrasy for want of a name to describe an unknown something, is clearly an underlying pathological relation of curative drug action, and is manifested by the over action of this curative drug in many instances.

The richest field of drug proving is found in provers with the peculiar idiosyncrasy for certain drugs. Hence the value of potentized drugs for proving, although only a few of our large number of provers bring out symptoms. The continued taking of potentized drugs develops a susceptibility to certain drugs, and such provers become better after several attempts. I have observed that patients become more sensitive to the homœopathic remedy after several years continuing to take purely potentized medicines; while the taking of crude substances so phlegmatize the system that no fine symptoms will be evolved or felt. I have patients with whom I can develop the curative antagonism by the single dose of the highest hand-made potency, who, if more medicine were given, would become sick of the over-drug action. Then it is plainly to be seen that chronic and acute idiosyncrasies are present in the subject, and instead of a fault to be regretted in a patient, should be studied comprehensively in its relation to its expressions, viz., symptomatology. This is the beautiful and pleasant work of the Hahnemannian. We are not baffled but encouraged by the existence of this so-called idiosyncrasy, as by finding which we have gained a strong hold, in the way of information, upon the constitution of our patient. It may be his or her peculiarity and the guiding symptom to a curative selection.

